# Artificial Intelligence for Drug Safety Across the Lifecycle and Decision Type: A Scoping Review

**DOI:** 10.3390/ph19020334

**Published:** 2026-02-19

**Authors:** Tae Woo Kim, Sihyeon Park, Miryoung Kim

**Affiliations:** 1 College of Pharmacy, Sunchon National University, Suncheon 57922, Republic of Korea; 2 College of Pharmacy and Research Institute of Life and Pharmaceutical Sciences, Sunchon National University, Suncheon 57922, Republic of Korea

**Keywords:** pharmacovigilance, adverse reactions, artificial intelligence, decision making

## Abstract

**Background/Objectives**: Artificial intelligence (AI) is increasingly applied to drug safety evaluation, yet evidence is dispersed across lifecycle stages and tasks. This scoping review aimed to (1) map how AI supports safety- and treatment-related decision types across the drug lifecycle, and (2) examine evaluation strategies used to assess model reliability for clinical or regulatory use. **Methods**: Using Arksey and O’Malley’s framework, we searched a major database for studies published in the past decade that applied AI or machine learning to drug safety or medication-related decisions. After screening, we extracted data on lifecycle stage, decision type, AI methods, data sources, and evaluation strategies. A lifecycle–decision matrix was constructed to characterize application patterns. **Results**: AI applications were concentrated in real-world clinical care × patient-level safety prediction and post-marketing × safety surveillance, using EHRs, spontaneous reporting systems, and clinical text. Common methods included gradient boosting, deep neural networks, graph neural networks, and natural language processing models. This concentration reflects structural incentives favoring safety-oriented applications with readily available data and lower decision liability. Evidence for treatment optimization, regulatory decision modeling, and evidence synthesis was limited. Most studies used internal validation; external validation and real-world deployment were uncommon, indicating early methodological maturity and limited translational readiness. **Conclusions**: AI demonstrates strong potential to enhance drug safety—particularly in risk prediction and pharmacovigilance—but its use remains uneven across the lifecycle. By situating AI applications within explicit lifecycle stages and decision contexts, this review clarifies where progress has advanced, where translation has stalled, and why these gaps persist. Limited external validation and minimal real-world testing constrain clinical and regulatory adoption. These findings suggest that external validation and real-world testing may contribute to further advances in AI for drug safety.

## 1. Introduction

Adverse drug reactions (ADRs) remain a major source of preventable harm worldwide, contributing to substantial morbidity, mortality, and healthcare utilization [1,2]. Traditional pharmacovigilance systems—especially spontaneous reporting systems (SRSs)—provide essential safety information but are limited by under-reporting, delayed signal detection, reporting biases, and difficulty capturing evolving risk profiles in real-world settings [3,4].

Pharmacoepidemiology has contributed significantly to post-marketing safety assessment by enabling systematic evaluation of drug exposure and outcomes at the population level [3,4,5]. The growing availability of real-world data (RWD)—including electronic health records (EHRs), administrative claims, and registries—offers new opportunities to analyze drug utilization patterns and safety outcomes [6,7]. However, conventional statistical methods often struggle with high-dimensional covariates, nonlinear associations, longitudinal trajectories, and rare adverse events, limiting their ability to generate timely and actionable insights for drug safety decision-making [8,9,10,11].

Against this backdrop, artificial intelligence (AI) and machine learning (ML) have emerged as promising tools to enhance drug safety evaluation and support medication-related decisions [12,13,14,15,16,17]. AI techniques are increasingly used to predict patient-level ADR risks, detect emerging safety signals, identify high-risk subgroups, and extract safety information from unstructured clinical and biomedical text. Early evidence suggests that AI can meaningfully complement traditional pharmacovigilance workflows and enable more precise and proactive medication management [18].

Despite growing interest, the literature remains fragmented. Existing reviews often emphasize algorithmic performance or molecular toxicity prediction, while giving less attention to issues such as clinical applicability, workflow integration, or alignment with regulatory expectations [18]. Recent syntheses, such as Toni et al. (2024), provide useful overviews of ML-based side effect prediction but largely focus on technical aspects and do not systematically assess how AI contributes to decision-making across the drug lifecycle [18].

A scoping review is well-suited to address this gap by mapping an emerging and heterogeneous evidence base [19,20,21]. Importantly, in rapidly evolving fields such as AI for drug safety, the key challenge is not only identifying where methods have been applied, but understanding how their maturity, validation practices, and decision relevance differ across contexts. In the context of AI for drug safety, studies vary widely in data sources, tasks, modeling approaches, and evaluation strategies, underscoring the need for a structured synthesis.

This review aims to provide a comprehensive overview of how AI methods are applied across the drug lifecycle to support key safety- and treatment-related decisions. Rather than focusing on individual model performance, the review uses a lifecycle–decision framework to examine patterns of application, identify structural gaps in translation, and assess how closely existing AI approaches align with clinical, pharmacovigilance, and regulatory decision needs. By organizing current evidence within a unified lifecycle–decision framework, it identifies where AI applications are most developed, where gaps remain, and why progress toward routine clinical and regulatory implementation has been uneven, thereby informing future research directions in pharmacoepidemiology and pharmacovigilance.

## 2. Materials and Methods

This scoping review was conducted using Arksey and O’Malley’s methodological framework, guided by PRISMA-ScR reporting standards, to systematically map how AI is currently applied across the drug lifecycle [21,22] (see PRISMA-ScR Checklist in the Supplementary Materials). The review protocol was prospectively registered in PROSPERO (1230579). The review was designed to address two core research questions:

(1) How is AI being applied across different stages of the drug lifecycle to support key safety- and treatment-related decision types, and what patterns emerge in tasks, data types, and AI methods within each lifecycle–decision domain?

(2) What evaluation strategies are used to assess AI model reliability, and to what extent do existing studies demonstrate readiness for clinical or regulatory use?

### 2.1. Literature Search and Study Selection

A structured literature search was conducted in PubMed using three concept groups: AI/ML methods (e.g., “artificial intelligence,” “machine learning,” “deep learning,” “neural networks,” “natural language processing”); decision or evaluation context (e.g., “decision support,” “clinical decision,” “dose optimization,” “patient selection,” “benefit–risk,” “risk prediction”); and drug lifecycle domains (e.g., “drug discovery,” “lead selection,” “dose selection,” “clinical trial,” “real-world data,” “pharmacoepidemiology,” “health technology assessment”). The three groups were combined using Boolean logic, yielding a comprehensive set of publications relevant to AI-supported drug lifecycle decisions (see Search Terms in the Supplementary Materials). Studies passing this stage proceeded to full-text review, where the inclusion criteria were applied more rigorously, focusing on whether the article developed or evaluated an AI model for drug-related safety, treatment optimization, or decision-making within any lifecycle stage. Full-text screening resulted in the final set of studies included in this review.

#### 2.1.1. Inclusion Criteria

Studies were included if they met all of the following conditions:

1. AI/ML contribution to drug safety: The study applied an AI or ML model that directly supported drug safety, pharmacovigilance, or medication-related risk assessment.
2. Decision relevance: The model generated outputs that could meaningfully aid medication safety, such as predicting ADRs, detecting safety signals, stratifying high-risk patients, or supporting treatment or regulatory decisions.
3. Regulatory or clinical applicability: The study referenced real-world clinical use, pharmacovigilance relevance, or potential regulatory/HTA utility.
4. Data relevance: The study used data sources connected to medication safety (e.g., EHR, claims, SRSs, registries, biomedical text, drug labels, social media, knowledge graphs). Studies leveraging social media data for AI-based analyses of adverse drug reactions or pharmacovigilance were also included, reflecting their use as complementary sources to spontaneous reporting systems.
5. Search-term relevance (specificity enhancement): To improve specificity, only studies whose title or abstract contained at least one of the predefined search keywords were included.
6. Availability: Full text was available for detailed review.
7. Date range: Published within the past 10 years.

#### 2.1.2. Exclusion Criteria

Studies were excluded if they did not demonstrate a direct and substantive connection to drug safety or pharmacovigilance. Research was omitted when it focused exclusively on laboratory-based or molecular-level modeling without linkage to clinical outcomes, medication-use patterns, or safety-related decision-making. We also excluded papers that presented algorithmic or methodological advances without clear implications for pharmacovigilance, clinical risk prediction, or regulatory assessment. Studies centered solely on biological mechanisms, as well as reviews, commentaries, protocols, conference abstracts, and papers reporting only descriptive summaries of side effects without predictive or decision-support relevance, were not eligible for inclusion.

### 2.2. Data Extraction

A structured template was used to extract study characteristics, therapeutic focus, lifecycle stage, decision type, data type, AI methodology, and evaluation metrics. Each study was assigned to one or more lifecycle stages and decision types based on its objectives and analytic framework.

### 2.3. Classification

To organize the diverse AI applications identified in the included studies, we conducted a comprehensive synthesis of extracted information across tasks, data types, and methodological characteristics. Through iterative categorization, we developed a structured classification framework that distinguishes both drug lifecycle stages and decision types. This framework was designed to capture meaningful differences in the purpose, data context, and operational relevance of AI models while ensuring that categories remain interpretable and mutually distinct.

Using this classification system, each study was systematically mapped onto one or more appropriate lifecycle–decision domains. This mapping was then visualized using a heatmap, which allowed us to identify lifecycle–decision domains with concentrated AI activity as well as those with limited supporting evidence. All records were managed using EndNote 21, and data extraction and categorization were performed in Microsoft Excel. After assigning each included study to its corresponding lifecycle–decision cell, we generated row-level count data representing the distribution of studies across the matrix. These structured data were then used to produce a heatmap visualization, created with assistance from ChatGPT-5.1.

### 2.4. Evaluation Strategy Classification

To understand how artificial intelligence is being used across diverse drug safety and treatment-related decision contexts, we categorized the AI methods and evaluation approaches extracted from each study according to their functional characteristics. Particular attention was given to whether studies reported procedures relevant to reproducibility and model validity, and how these procedures were implemented.

## 3. Results

### 3.1. Overview of Included Studies

**Figure 1:**
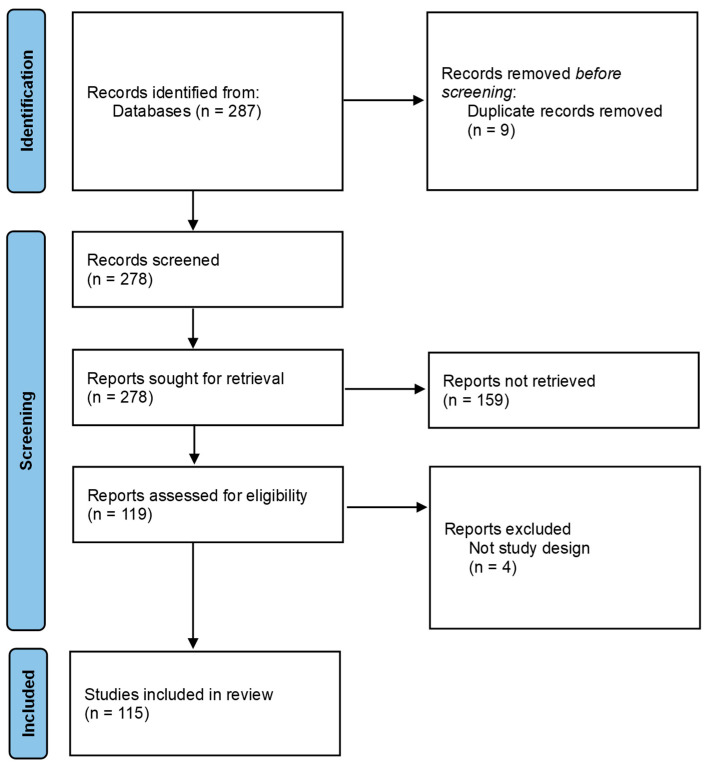
PRISMA Flow diagram.

The literature search initially identified 287 records relevant to AI applications in drug safety and therapeutic decision-making. After removing 9 duplicates, 278 unique studies underwent title and abstract screening. A total of 119 studies proceeded to full-text assessment, and after applying the second-stage exclusion criteria, 115 studies met all eligibility requirements and were included in the final scoping review (Figure 1).

#### 3.1.1. Year of Publication Distribution

The 115 included studies were published between 2016 and 2025, with a pronounced acceleration in recent years. 31 studies were published annually before 2021 [23,24,25,26,27,28,29,30,31,32,33,34,35,36,37,38,39,40,41,42,43,44,45,46,47,48,49,50,51,52,53]. Beginning in 2022, publication volume increased sharply (22 studies in 2022 [14,54,55,56,57,58,59,60,61,62,63,64,65,66,67,68,69,70,71,72,73,74]; 11 in 2023 [13,75,76,77,78,79,80,81,82]), followed by a substantial surge in 2024 (26 studies [15,17,83,84,85,86,87,88,89,90,91,92,93,94,95,96,97,98,99]) and 2025 (25 studies [12,16,100,101,102,103,104,105,106,107,108,109,110,111,112,113,114,115,116,117,118,119,120]). More than 70% of all included studies were published within the last four years, reflecting the rapid expansion of AI applications in pharmacoepidemiology, drug safety surveillance, and treatment decision support.

#### 3.1.2. Clinical and Therapeutic Areas

All studies covered a wide range of therapeutic domains, with notable concentrations in:

- Oncology (e.g., chemotherapy toxicity, treatment response, immune-related ADRs) [14,43,66,72,89,118,121];
- Cardiovascular and metabolic diseases (e.g., QT prolongation, Drug-induced liver injury, diabetes treatment optimization) [34,38,42,47,101,103,122];
- Infectious diseases, including tuberculosis and antibiotic-related toxicities [48,113];
- Autoimmune and inflammatory diseases (e.g., ulcerative colitis biologics, dermatologic immune reactions [67,118,123]);
- General pharmacovigilance contexts, where no single disease was the focus (e.g., signal detection in adverse event reporting system database, text-based ADR extraction, multi-label ADR prediction) [33,34,35,36,39,41,56,57,59,63,79,84,97,98,102,112,124].

Across these areas, oncology and cardiometabolic conditions were most frequently represented, reflecting both high clinical burden and robust availability of structured and unstructured patient data. A substantial subset of studies—particularly those based on SRSs or biomedical text—focused on drug-level or class-level safety evaluation rather than disease-specific populations.

Overall, all studies encompass a diverse and rapidly evolving evidence base, spanning multiple data modalities, methodological innovations, and clinical application domains across the drug lifecycle.

### 3.2. Lifecycle and Decision Type Mapping of AI Applications

We systematically categorized each AI application according to (1) the stage of the drug lifecycle in which the model was applied and (2) the primary decision type the model was intended to support. This dual-axis classification framework enabled a structured synthesis of a highly heterogeneous body of literature, and provided an overarching view of how AI is currently being used to inform drug safety and treatment-related decision-making (Table 1). The ordering of lifecycle stages reflects the hierarchy and scope of decision-making rather than strict chronological sequencing. L5 (Regulatory/HTA/Market Access) represents system-level decisions that integrate evidence generated across earlier stages, rather than a temporally subsequent phase.

First, all studies were mapped to one of five lifecycle stages. These stages reflect the continuum from early drug design to real-world utilization, post-marketing surveillance, and regulatory decision-making. The majority of studies clustered within L1 [23,25,26,27,28,31,34,38,40,42,45,54,58,64,67,68,70,73,76,82,85,87,88,91,93,95,96,99,104,106,107,108,109,110,111,112,117,125] and L3–L4 [12,13,14,15,16,17,24,29,30,32,33,35,36,37,39,41,44,46,47,48,49,50,51,52,53,55,56,57,59,60,61,63,65,66,69,71,72,74,77,78,79,80,81,83,84,86,89,90,92,97,98,100,101,102,103,105,113,114,118,119,120,123,124,126,127,128,129,130,131], where AI was frequently used to support clinical prescribing decisions and large-scale pharmacovigilance tasks using RWD. Only a small subset of studies (*n* = 4) focused on L5, indicating that applications directly supporting regulatory or Health Technology Assessment (HTA) processes remain relatively underdeveloped [30,75,94,127]. Second, each study was assigned to one of six decision types. Most studies addressed D1 [12,13,14,15,16,17,23,24,25,27,28,38,40,42,45,46,48,50,54,55,58,60,67,68,70,71,72,73,74,76,78,80,82,85,86,87,88,92,95,99,100,101,103,104,106,107,108,109,110,111,112,113,114,118,120,125,126,129,131] or D4 [26,32,34,35,36,39,41,44,45,49,53,56,57,59,61,63,64,69,75,77,79,81,82,83,84,90,96,97,98,102,112,124,132]. Applications focused on treatment optimization (D3) [29,31,32,37,43,47,51,52,66,116,117,123,128] and clinical effectiveness/prognosis (D2) [31,37,43,47,48,52,65,66,89,91,93,116,119,123,125,130,133] were moderately represented, while no studies addressed evidence synthesis & decision modeling for market access (D5), and Policy/Strategy/Framework Design (D6) were rarely the primary objectives [30,62,94,105,115,127].

### 3.3. Lifecycle–Decision Type Matrix

**Figure 2:**
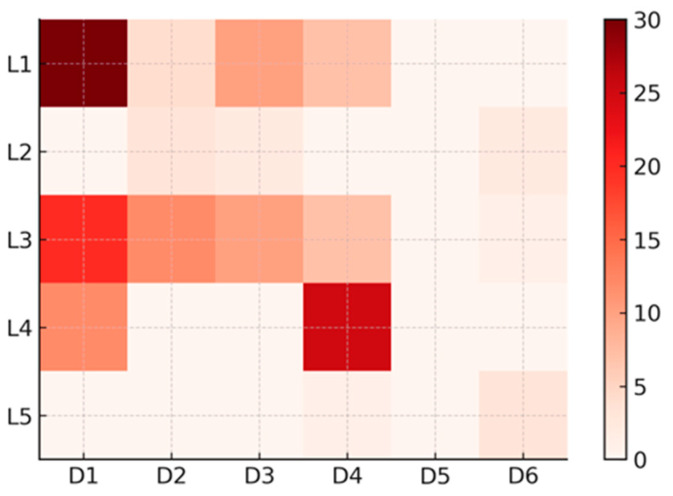
Heatmap of Artificial Intelligence Applications Across Drug Lifecycle Stages and Decision Types.

Using these classifications, all studies were mapped onto a Lifecycle × Decision matrix, which highlights the distribution of evidence across the two dimensions (Figure 2).

This mapping revealed clear patterns:

- Early-stage studies (L1) focused mainly on mechanistic or structural safety predictions (D1) and treatment optimization tasks (D3);
- Clinical-stage research (L2) targeted prognostic modeling (D2) and dosage/response optimization;
- Real-world care (L3) emphasized patient-level safety prediction (D1) and clinical decision support (D3);
- Post-marketing studies (L4) overwhelmingly concentrated on large-scale signal detection and surveillance (D4);
- Regulatory/HTA applications (L5) were limited but centered on D5 and D6 decision types.

### 3.4. Summary of AI Applications Across Lifecycle–Decision Domains

Table 2, Table 3, Table 4, Table 5 and Table 6 summarize how AI has been applied across different stages of the drug lifecycle and decision types, highlighting the core tasks, data sources, and AI methods observed in the included studies. AI applications varied widely across the stages of the drug lifecycle. In the L1, most studies focused on early safety prediction, estimation of drug response, and optimization of chemical structures or drug combinations. These models drew on chemical and biological assays, drug–target or drug–ADR networks, and omics datasets. Techniques such as GNNs, multi-label deep learning, and generative modeling were commonly used, reflecting the exploratory and data-rich nature of this research phase (Table 2).

Applications in L2 were less frequent but targeted more specific tasks, such as predicting tumor growth, cognitive decline, or treatment response. A few studies also examined regulatory-relevant questions, including the likelihood of drug approval. These analyses typically relied on imaging, longitudinal biomarkers, and psychometric measures, using clustering approaches, survival models, and ensemble ML methods (Table 3).

AI use in L3 was broader and more diverse. Many studies aimed to predict patient-level safety risks, clinical outcomes, or treatment effectiveness using EHR data—vital signs, laboratory results, medication histories, electrocardiograms, and pharmacogenomic profiles. NLP-based extraction of ADRs from clinical text also appeared frequently, showing how AI can support routine documentation and pharmacovigilance activities within healthcare settings (Table 4).

L4 represented one of the most active areas. Studies used SRSs, social media text, and hospital data to detect safety signals or estimate population-level ADR risks. Transformer-based NLP, enhanced disproportionality methods, and multimodal deep learning were widely adopted. A smaller group of studies explored converting guideline text into structured safety rules, signaling interest in automating safety oversight (Table 5).

By contrast, AI applications in L5 were still emerging. These studies focused mostly on predicting label-update or HTA adoption decisions and on extracting safety-relevant information from regulatory documents. Transformer-based NLP and explainable ML played a central role in these efforts (Table 6).

Taken together, the literature shows strong concentration of AI work in patient-level safety prediction (D1) and post-marketing signal detection (D4), particularly in discovery/preclinical, clinical care, and post-marketing settings. Fewer studies addressed treatment optimization (D3) or effectiveness prediction (D2), and almost none engaged with evidence synthesis for market access (D5). This pattern suggests areas where AI methods are maturing, as well as clear gaps where future development is needed.

### 3.5. Model Evaluation Strategies and Reliability

Across the 115 included studies, evaluation practices showed a clear concentration around internal validation and benchmark comparison, with far fewer examples of external testing or real-world deployment (Table 7).

Internal validation was almost universal, as most studies relied on train–test splits, time-based partitioning, or k-fold cross-validation within a single dataset. These approaches ensured basic model stability but offered limited insight into generalizability beyond the development context. Similarly, benchmark comparisons were conducted in the majority of studies, typically contrasting AI models with disproportionality analyses, logistic regression, or other ML baselines. While these comparisons demonstrated performance gains, they largely reflected technical superiority rather than readiness for clinical adoption.

Only a small subset of studies (n = 16) conducted external validation using independent datasets from different hospitals, registries, or geographic regions [13,17,43,60,71,73,78,80,89,93,100,101,126]. These studies provided stronger evidence of reproducibility but remained the exception rather than the norm. External validation was more common in research using structured EHR or claims data, whereas studies relying on SRSs or molecular datasets rarely extended evaluation beyond the original source.

Real-world or prospective deployment was particularly rare. Only two studies integrated AI models into an active clinical workflow or EHR system, underscoring a substantial translational gap between algorithm development and practical implementation [39,98]. No study reported regulatory-grade validation or formal assessment aligned with HTA or pharmacovigilance decision-making standards.

Overall, the landscape reveals a heavy methodological emphasis on internal performance metrics and a relative shortage of rigorous validation strategies that assess robustness, transportability, or operational readiness. These findings highlight the need for more systematic external validation, prospective evaluation, and reporting practices that reflect the requirements of real-world clinical and regulatory environments.

## 4. Discussion

This scoping review demonstrates that the maturity and translational readiness of AI applications for drug safety are not uniform, but systematically differ by drug lifecycle stage and decision type. While methodological innovation has advanced rapidly—particularly in early discovery and post-marketing surveillance contexts—progress toward routine clinical use varies across application domains. By organizing existing evidence within a lifecycle–decision framework, this review highlights not only where AI development has been most active, but also where additional validation and implementation efforts may further support progress in AI-enabled drug safety and pharmacovigilance.

While several prior reviews have examined the role of AI in drug safety, their scope has typically been narrowed—focusing on particular methodological families, single application domains, or high-level conceptual opportunities without explicitly situating these applications within the decision-making processes they are intended to support [18,134,135]. A scoping review of ML-based side effect prediction offers valuable methodological detail but remains largely anchored in preclinical and molecular toxicity modeling, with limited relevance to pharmacoepidemiologic data or real-world pharmacovigilance workflows [18,134]. Likewise, a review of AI in clinical pharmacy highlights potential benefits for medication safety but does not assess how AI methods align with specific decision categories or whether these approaches demonstrate sufficient methodological maturity for real-world implementation [135]. In contrast, the present review synthesizes evidence across lifecycle stages, decision types, data modalities, and evaluation practices, thereby enabling a structural assessment of where AI applications are most developed, where translation has stalled, and why these gaps persist across different decision contexts.

The lifecycle–decision mapping showed that AI activity was most pronounced in the preclinical in silico domain for patient-level safety prediction (L1–D1), where graph-based, embedding, and multimodal models were widely used to predict ADRs, DDIs, and off-target effects [23,25,27,28,38,40,42,45,54,58,67,68,70,73,76,82,85,87,88,95,99,104,106,107,108,109,110,111,112,125]. Substantial clustering was also observed in clinical care × patient-level safety prediction (L3–D1) [12,13,24,46,48,50,60,71,72,74,78,80,100,101,103,113,114,118,126,131] and post-marketing × safety surveillance (L4–D4) [32,33,35,36,39,41,44,49,53,57,59,61,63,69,77,79,81,83,84,90,97,98,102,124,132]. This concentration reflects key characteristics of drug safety and pharmacovigilance settings, including broad data availability, relatively well-defined safety signals, and established surveillance workflows that align well with AI-based prediction and prioritization. As a result, current AI development has been most advanced in safety-focused applications across early discovery and real-world pharmacovigilance. In contrast, Evidence synthesis, regulatory decision modeling, and policy-level applications (L5–D5/D6) were rarely represented, suggesting that these areas may benefit from further methodological development and clearer alignment between AI outputs and decision-making needs, rather than reflecting limited relevance for drug safety [30,62,94,115,127]. Overall, the lifecycle–decision matrix reveals an uneven but informative landscape, with robust development in safety-focused domains and opportunities for continued progress in higher-level evaluative and regulatory decision stage

Although methodological innovation was widespread, most studies remained at an early stage of maturity in terms of validation. Internal validation—typically cross-validation or train/test splits—was almost universal, whereas external validation was performed in only a small subset of studies [13,17,37,43,48,60,71,78,80,89,93,100,101,103,118,126]. This pattern can be understood in light of the practical characteristics of many drug-safety data sources, particularly spontaneous reporting systems, where independent validation datasets with stable denominators and adjudicated outcomes are often limited. Real-world deployment was even rarer, limited to a few examples involving NLP-based ADR extraction or automated pharmacovigilance triage [39,98]. Moreover, few studies assessed calibration, interpretability, fairness, or temporal robustness. Greater attention to these aspects may help strengthen confidence in model transportability and operational use, particularly in settings where AI outputs could inform safety-related decisions. From a drug safety perspective, expanded external validation and real-world evaluation may contribute to enhancing the reliability and practical relevance of AI models, complementing existing pharmacovigilance systems. To achieve meaningful clinical utility, AI models must demonstrate not only predictive accuracy but also credible validation, reliability across settings, and interpretability that supports safe decision-making [136,137]. Addressing these considerations may support the gradual translation of high-performing AI models into routine pharmacovigilance and clinical workflows. To support the translation of AI models into drug safety and pharmacovigilance practice, future studies may benefit from clearer evaluation and reporting practices. In particular, greater use of external or temporal validation, calibration assessment, and interpretable model outputs may enhance confidence in real-world applicability. Explicit alignment between AI outputs and pharmacovigilance decision contexts may further support responsible implementation.

This review has several notable strengths. It is one of the few studies to systematically organize AI applications using a lifecycle–decision framework, allowing a more integrated view of how AI contributes to drug safety across different stages of development, clinical use, and regulatory evaluation. By drawing on a decade of research and synthesizing heterogeneous evidence—from preclinical prediction models to post-marketing surveillance tools—the review offers a comprehensive landscape that captures both the breadth and the diversity of current AI approaches. Additionally, the structured categorization of data types, AI methods, and evaluation strategies provides a practical foundation for identifying methodological trends and assessing the maturity of different application domains. By emphasizing decision context and pharmacovigilance relevance rather than algorithmic novelty alone, this mapping approach helps clarify where AI has already demonstrated value for drug safety and where further evaluation efforts may support near-term impact. Accordingly, this framework may be useful for guiding future empirical studies, encouraging more consistent reporting practices, and informing methodological discussions in pharmacoepidemiology and pharmacovigilance.

Despite these strengths, several limitations should be acknowledged. As with any scoping review, the focus was on breadth rather than depth; individual model performance, statistical rigor, and domain-specific nuances were not evaluated in detail. This approach was intentional and aligned with the aim of mapping an emerging and heterogeneous evidence base, rather than adjudicating the quality of individual models. The review also relied primarily on studies published in the past ten years and indexed in major databases, which may have excluded relevant work in grey literature, industry reports, or non-English publications. Moreover, the heterogeneity of reporting across studies—particularly regarding validation strategies, data preprocessing, and performance metrics—limited the ability to compare models directly or assess best practices. At the same time, this heterogeneity represents an informative finding, reflecting the evolving nature of evaluation practices and the absence of shared standards across AI applications in drug safety. Finally, because few studies provided real-world implementation evidence, conclusions about the translational readiness of AI tools necessarily remain provisional. Rather than a limitation unique to this review, this observation highlights an important opportunity for future empirical research and methodological development in pharmacovigilance. These considerations underscore the value of more standardized evaluation frameworks, richer methodological reporting, and future systematic reviews focused on narrower aspects of AI-driven drug safety.

Overall, the results indicate that AI has considerable potential to advance drug safety evaluation and support decision-making across the lifecycle, although its current impact varies across application domains. By synthesizing evidence within a lifecycle–decision framework, this review clarifies the structural factors shaping where AI has progressed most rapidly and where further validation and real-world evaluation may help extend its contribution. By providing a structured overview of current applications and identifying unmet needs, this review offers a contextual foundation for future methodological development and for AI systems intended to complement and strengthen pharmacovigilance practice.

## Figures and Tables

**Table 1:** Classification Framework for Mapping Artificial Intelligence Applications by Drug Lifecycle Stage and Decision Type.

**Lifecycle Stage**		
L1	Discovery/Preclinical/In silico Design	Early discovery, computational design, molecular screening, preclinical modeling
L2	Clinical Development & Trial Design	Clinical trials, protocol optimization, outcome prediction during development
L3	Prescribing/Patient Management	Individual-level treatment decision-making in real-world care
L4	Post-marketing Safety & Effectiveness	Population-level RWE safety studies, surveillance systems, large-scale monitoring
L5	Regulatory/HTA/Market Access	Benefit–risk assessment, label change, HTA review support
**Decision type**		
D1	Patient-level Safety Risk Prediction	Predicting ADRs, toxicity, DILI, organ-specific injury risk, high-risk patient stratification
D2	Effectiveness/Prognosis Prediction	Treatment response, disease progression, survival, symptom trajectory
D3	Treatment Choice/Dose Optimization	Drug selection, regimen ranking, dose setting, personalized medicine
D4	Safety Signal Detection & Surveillance	Disproportionality, early detection of unexpected ADRs, large-scale PV monitoring
D5	Evidence Synthesis & Decision Modeling for Market Access	Approval prediction, label change modeling, HTA evaluation
D6	Policy/Strategy/Framework Design	Regulatory frameworks, PV system design, policy planning

Abbreviations: ADR, Adverse Drug Reaction; DILI, Drug-Induced Liver Injury; HTA, Health Technology Assessment; PV, Pharmacovigilance; RWE, Real-World Evidence.

**Table 2:** Summary of AI Applications Across Lifecycle Stage 1 and Decision Types.

		Key Tasks	Data	AI Methods	Reference
L1	D1	Predict drug–ADR associations, organ toxicity, off-target effects Detect high-risk DDIs, multi-label ADR classification Build knowledge graph or network-based safety screening tools	Chemical structure, biological assays Drug–target, drug–ADR networks Omics, phenotype embeddings	GNN Multi-label DNN, embedding models, matrix factorization Contrastive learning/multi-task learning	[23,25,27,28,38,40,42,45,54,58,67,68,70,73,76,82,85,87,88,95,99,104,106,107,108,109,110,111,112,125]
	D2	Predict drug synergy, target sensitivity, biomarker-driven response Rank effective drug combinations in cell/animal models	Omics, biochemical assays, cell line panels Chemical similarity, pharmacological networks	Generative models (VAE, reinforcement learning) GNN, multi-modal learning, contrastive learning	[31,91,93,125]
	D3	Optimize chemical structure (e.g., BBB permeability, KRAS inhibitor design) Predict optimal drug combinations and DDI-safe regimens	Compound structures, molecular graphs DDI networks, phenotype data	GNN, contrastive learning, multi-modal deep networks, generative models (VAE/RL)	[31,38,76,95,99,106,108,109,111,117]
	D4	Literature/biomedical-text mining for ADR relations KG-based unknown ADR discovery Early signal identification using text + network fusion	MEDLINE, Drug–ADR knowledge graphs Biomedical corpora	Multi-task NLP, attention-based relation extraction Weak supervision, KG-enhanced models	[26,34,45,64,82,96,112]
	D5	NA	NA	NA	
	D6	NA	NA	NA	

Abbreviations: ADR, Adverse Drug Reaction; DDI, Drug–Drug Interaction; GNN, Graph Neural Network; DNN, Deep Neural Network; NLP, Natural Language Processing; KG, Knowledge Graph; BBB, Blood–Brain Barrier; VAE, Variational Autoencoder; RL, Reinforcement Learning; NA, Not Applicable.

**Table 3:** Summary of AI Applications Across Lifecycle Stage 2 and Decision Types.

		Key Tasks	Data	AI Methods	Reference
L2	D1	NA	NA	NA	
	D2	Predict tumor dynamics, overall survival clusters Forecast cognitive/functional decline in AD trials	Clinical trial imaging, longitudinal biomarkers Questionnaire/psychometric scores	Clustering + survival analysis Regression ML (RF, SVR, etc.)	[43,116,133]
	D3	Stratify trial participants for optimal treatment arms Predict responder vs. non-responder	Trial biomarker sets, imaging data	Regression ML, ensemble models	[43,116]
	D4	NA	NA	NA	
	D5	NA	NA	NA	
	D6	Predict drug approval probability/clinical development failure Identify bias in trial data and debias outcome (regulatory relevance)	Citeline/pipeline data, drug properties	Debiasing VAE, GNN + molecular embeddings	[62,115]

Abbreviations: AD, Alzheimer’s Disease; D1–D6, Decision Types 1–6; GNN, Graph Neural Network; ML, Machine Learning; RF, Random Forest; SVR, Support Vector Regression; VAE, Variational Autoencoder; NA, Not Applicable.

**Table 4:** Summary of AI Applications Across Lifecycle Stage 3 and Decision Types.

		Key Tasks	Data	AI Methods	Reference
L3	D1	Predict nephrotoxicity, hepatotoxicity, hematologic toxicity Predict QT prolongation, thrombocytopenia, chemotherapy toxicities	EHR (vitals, labs, medications), PGx, ECG Multi-institution CDM networks	Tree-based ML (XGBoost, RF), LSTM, ANN Federated learning, AutoML	[12,13,24,46,48,50,60,71,72,74,78,80,100,101,103,113,114,118,126,131]
	D2	Predict clinical response or treatment persistence (UC biologics, oncology) Predict radiographic progression (axSpA), antidepressant adherence	Clinical cohorts, imaging, labs EHR sequences	Clustering + ML, LightGBM RNN/sequence models	[37,47,48,52,65,66,89,116,119,123,130,133]
	D3	Personalized dose selection: warfarin, anesthesia, digoxin Therapy ranking (UC biologics, psychiatric therapy optimization)	EHR, PGx, time-series data, surgery monitoring signals	ANN, CNN-BiLSTM fusion Gradient boosting, regression ML	[29,37,46,47,48,51,52,65,66,89,116,119,123,128,130,133]
	D4	Extract ADR mentions from EHR text Detect ADE relations using BERT or LLM Improve allergy documentation systems	Clinical narratives, discharge summaries N2c2 corpora, institution-specific text datasets	BERT-based NER/RE, rule-based NLP LLM-assisted extraction, hybrid models	[39,49,56,61,97,98,124]
	D5	NA	NA	NA	
	D6	Conceptual framework for individualized AI-assisted pharmacotherapy Identify implementation barriers and evaluation strategies	Clinical trial DB, Molecular graphs	Debiasing Variational Autoencoder, attention-based deep GNN	[105]

Abbreviations: ADE, Adverse Drug Event; ADR, Adverse Drug Reaction; ANN, Artificial Neural Network; BERT, Bidirectional Encoder Representations from Transformers; BiLSTM, Bidirectional Long Short-Term Memory; CDM, Common Data Model; CNN, Convolutional Neural Network; ECG, Electrocardiogram; EHR, Electronic Health Record; GNN, Graph Neural Network; LSTM, Long Short-Term Memory; LLM, Large Language Model; ML, Machine Learning; NLP, Natural Language Processing; PGx, Pharmacogenomics; QT, QT interval; RF, Random Forest; RNN, Recurrent Neural Network; NA, Not Applicable.

**Table 5:** Summary of AI Applications Across Lifecycle Stage 4 and Decision Types.

		Key Tasks	Data	AI Methods	Reference
L4	D1	Predict patient-level ADRs from FAERS + EHR hybrid data Teratogenicity, hematologic toxicity, herb-induced reactions Pregnancy-related ADR severity prediction	FAERS, national PV databases, pregnancy registries Hospital EHR	GNN, boosting models, deep ensembles Hybrid multimodal models	[14,15,16,17,55,74,86,92,120,126,129,131]
	D2	NA	NA	NA	
	D3	Convert textual guidelines into machine-readable safety rules	Clinical guideline text	NLP mapping, terminology-based extraction	[32]
	D4	Detect ADR signals from FAERS, JADER, VigiBase-like datasets Social media pharmacovigilance (Twitter, forums, multilingual text) Automated ICSR triage, disproportionality + ML fusion Document-level ADE extraction for automated PV workflows	SRS (FAERS, JADER), social media, EHR text Company PV databases	Disproportionality + ML pipelines BERT, LSTM, transformer-based text mining Transfer learning, attention mechanisms	[44,49,53,124,132]
	D5	NA	NA	NA	
	D6	Predict HTA/label-update decisions Map regulatory workflows with AI augmentation Design national PV modernization frameworks	HTA reports, PI revision history, regulatory documents	NLP classifiers, explainable ML Conceptual system design	[30,127]

Abbreviations: ADR, Adverse Drug Reaction; ADE, Adverse Drug Event; EHR, Electronic Health Record; FAERS, FDA Adverse Event Reporting System; PV, Pharmacovigilance; GNN, Graph Neural Network; HTA, Health Technology Assessment; ICSR, Individual Case Safety Report; JADER, Japanese Adverse Drug Event Report database; LSTM, Long Short-Term Memory; ML, Machine Learning; NLP, Natural Language Processing; SRS, Spontaneous Reporting System; NA, Not Applicable.

**Table 6:** Summary of AI Applications Across Lifecycle Stage 5 and Decision Types.

		Key Tasks	Data	AI Methods	Reference
L5	D1	NA	NA	NA	
	D2	NA	NA	NA	
	D3	NA	NA	NA	
	D4	Automatically identify and extract safety-related statements from FDA drug labels	FDA labeling text	Transformer-based NLP models	[75]
	D5	NA	NA	NA	
	D6	Predict HTA adoption (CONITEC), label-change likelihood (Japan PI) Regulatory intelligence for safety-driven decisions	HTA summaries, PI revision metadata, ADR case aggregates	NLP, SVM/XGBoost, risk modeling Explainable feature analysis	[30,94,127]

Abbreviations: ADR, Adverse Drug Reaction; HTA, Health Technology Assessment; NLP, Natural Language Processing; PI, Prescribing Information; SVM, Support Vector Machine; NA, Not Applicable.

**Table 7:** Model Evaluation Strategies and Reliability Assessment.

Evaluation Strategy	Definition	Examples of Methods	References
Internal validation	Model is evaluated only within the same dataset using cross-validation or random splits.	Train/test split, Time-split, K-fold cross-validation, Hold-out test set	Nearly all studies (n = 113)
Benchmark comparison	Model performance is compared against established baselines or traditional statistical methods.	vs. Disproportionality analysis, Logistic regression, Scoring-based models, Other ML/DL baselines	Nearly all studies (n = 109)
External validation	Model is validated on an independent dataset from a different hospital, region, registry, or cohort.	Cross-dataset validation	[13,17,43,60,71,73,78,80,89,93,100,101,126]
Real-world deployment	Model is tested within an active clinical workflow or real-time environment.	EHR-integrated system	[39,98]

## Data Availability

No new data were created or analyzed in this study. Data sharing is not applicable to this article.

## Supplementary Materials

The following supporting information can be downloaded at: https://www.mdpi.com/article/10.3390/ph19020334/s1, Table S1: Preferred Reporting Items for Systematic reviews and Meta-Analyses extension for Scoping Reviews (PRISMA-ScR) Checklist. Table S2: Search term.
